# Ebola: Ten years later—Lessons learned and future pandemic preparedness

**DOI:** 10.1371/journal.pgph.0003662

**Published:** 2024-09-26

**Authors:** Krutika Kuppalli

**Affiliations:** 1 Department of Medicine, University of Texas Southwestern, Dallas, Texas, United States of America; 2 School of Public Health, University of Texas Southwestern, Dallas, Texas, United States of America; PLOS: Public Library of Science, UNITED STATES OF AMERICA

In early December 2013, a 2-year-old boy in the remote village of Meliandou, Guinea fell ill with a mysterious disease and succumbed to the illness a few days later [1]. The disease rapidly spread, resulting in 49 cases and 29 deaths before being identified as the Zaire strain of the Ebola virus and officially declared an outbreak on March 23, 2014 [2]. Over the ensuing months, the outbreak spread to neighboring Liberia and Sierra Leone, with cases also emerging in Senegal, Nigeria, Mali, the United States and Europe [3]. On August 8, 2014, the World Health Organization (WHO) Director-General declared the outbreak a Public Health Emergency of International Concern (PHEIC), the highest global health alert, signifying the outbreak posed a public health risk to other Member States and necessitated a coordinated international response [4]. Despite global efforts to control the outbreak, it lasted for over two years, resulting in over 28,000 cases and more than 11,000 deaths by the time it was declared over on June 9, 2016 [3]. This crisis exposed significant weaknesses in global health systems, prompting a re-evaluation of pandemic preparedness and response strategies. A decade later, it is essential to reflect on the lessons learned from the West Africa Ebola crisis and their impact on current and future pandemic preparedness efforts. These lessons are outlined using the Health Emergency Preparedness and Response (HEPR) architecture developed by WHO, focusing on strengthening five core health emergency components: collaborative surveillance, safe and scalable care, community protection, access to countermeasures, and emergency coordination (Fig 1) [5].

**Fig 1:**
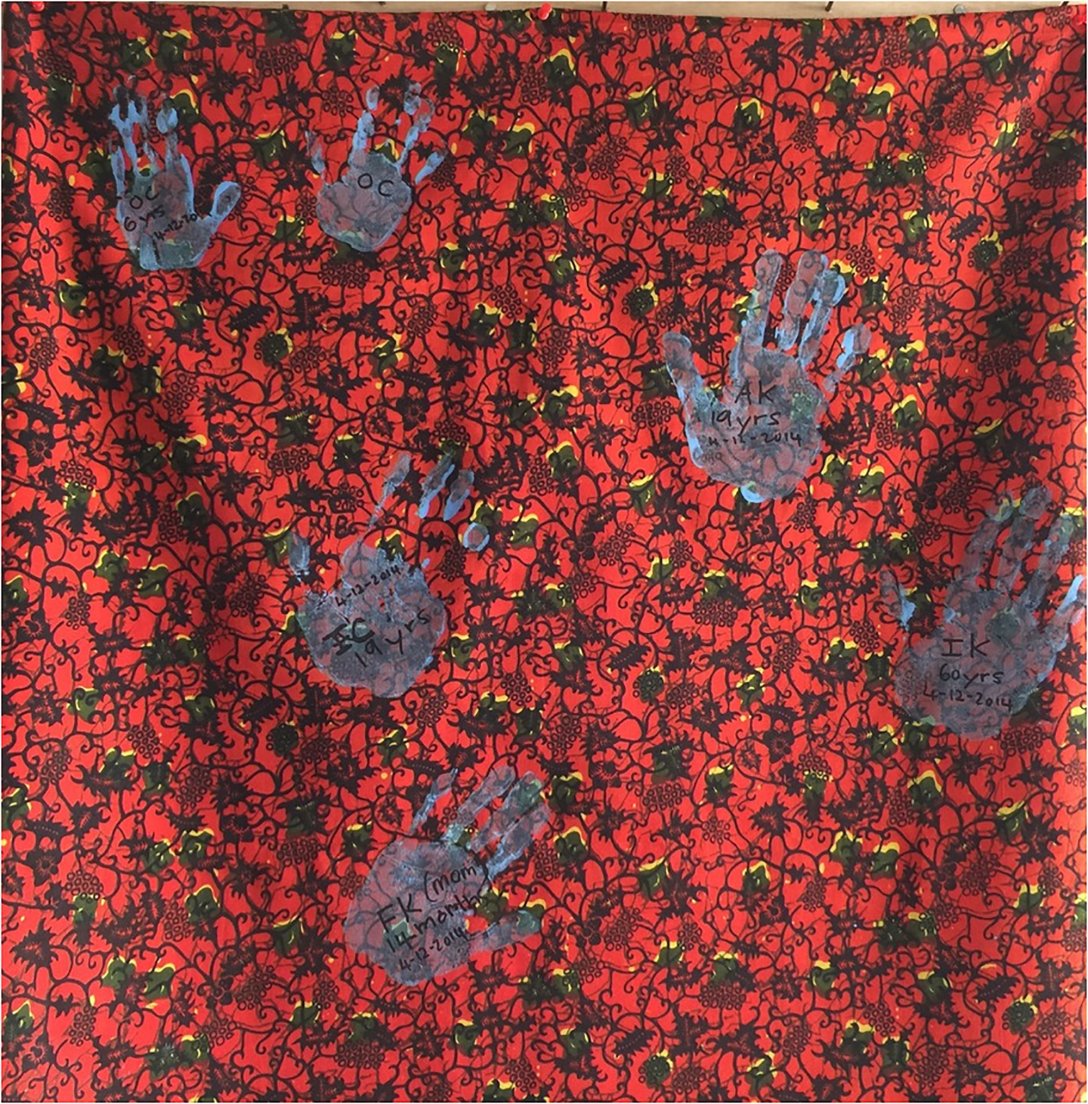
The author worked as the medical director of a large Ebola treatment unit in Sierra Leone during the 2014 West Africa Ebola outbreak, and invited survivors to put their handprint on this lapa. Survivors were invited to put their handprint on this lapa. Image credit: Krutika Kuppalli.

## Collaborative surveillance

Early detection and rapid response are critical in controlling outbreaks. During the Ebola epidemic, delays in recognizing initial cases in Guinea allowed the virus to spread unchecked [1]. The first case occurred in December 2013, but due to delays in recognition–largely because Ebola Virus Disease (EVD) had not been previously detected in this region–cases were not confirmed until late March 2014 [1]. By then, the outbreak had spread for over three months and was already out of control. Advances in diagnostic and surveillance systems, such as genomic sequencing and the use of technology for real-time data collection and analysis, have significantly improved our ability to detect and respond to outbreaks swiftly. This progress has led to more robust surveillance systems and quicker mobilization of resources and personnel during new outbreaks. The formation of rapid response teams that can be quickly deployed, along with the development of clear protocols—particularly for contact identification, education, and follow-up—are essential components of this strategy.

## Safe and scalable care

The Ebola outbreak highlighted the urgent need for robust health systems and optimized patient management. In the most affected countries, overwhelmed healthcare systems struggled to provide adequate care due to shortages of trained staff, medical supplies, testing infrastructure, logistical support, and financial resources [6]. These deficiencies hampered patient identification and treatment, facilitating the virus’s spread.

An indirect impact of the EVD outbreak was a significant setback in treating malaria, HIV/AIDS, and tuberculosis, resulting in an estimated 10,600 deaths in Guinea, Liberia, and Sierra Leone [7]. The influx of Ebola patients reduced essential health services like vaccinations, maternity care, and routine healthcare, leading to higher mortality rates [8]. The outbreak underscored the necessity for specialized training and support for healthcare workers handling infectious diseases and high-risk patients. Strict infection control practices, such as proper use of personal protective equipment (PPE), isolation protocols, and hygiene practices, were crucial in preventing nosocomial infections.

In response, international efforts focused on strengthening health systems, particularly in vulnerable regions. This included improving healthcare facilities with triage protocols, training in infection prevention and control (IPC), ensuring essential medical supplies like PPE were available, and enhancing diagnostic capabilities. The crisis spurred innovations in clinical care, such as optimized supportive care, rapid diagnostic tests, and experimental treatments and vaccines [9]. These measures have had lasting impacts on infectious disease management and improved preparedness for future pandemics. A strong health system is the first line of defense against any infectious disease outbreak.

## Community protection

Challenges with community engagement hindered effective public health interventions during the 2014 Ebola outbreak [10]. Mistrust of health authorities and international aid workers, stemming from a history of neglect and the sudden influx of foreign medical personnel, exacerbated resistance to medical interventions [10]. Traditional cultural beliefs and practices, such as burial rituals involving washing and touching the deceased, facilitated the virus’s spread and were difficult to change due to their deep cultural significance [11]. Communication barriers, including language differences and low literacy levels, hampered the dissemination of public health messages, which were often misunderstood or failed to reach the intended audience [12]. Stigma associated with Ebola led to social isolation of patients and their families, deterring individuals from seeking medical help [11]. Instances of violence and hostility towards health workers, fueled by fear and misinformation, made it dangerous to operate in certain areas. Political and socioeconomic factors, such as weak governance, lack of infrastructure, and economic hardship, further strained the relationship between communities and health authorities. Effective communication strategies, culturally sensitive health education, and involving community leaders were essential in overcoming these challenges.

Consequently, future responses for Ebola and other pathogens have prioritized these approaches to enhance the uptake of public health measures. Building trust and fostering community cooperation have proven to be indispensable in managing health crises. Addressing these challenges in future outbreaks requires early and continuous engagement, developing culturally appropriate communication strategies, addressing stigma, and strengthening health systems and infrastructure to ensure a robust and effective response.

## Access to countermeasures

The Ebola outbreak accelerated research and development through regulatory review of vaccines, therapeutics, and diagnostic tools. The successful development and deployment of therapeutics such as ZMapp along with the chimpanzee adenovirus 3 vaccine (ChAd3-EBO-Z) and the recombinant vesicular stomatitis virus vaccine (rVSVΔG-ZEBOV-GP) Ebola vaccine in the middle of a health crises were major milestones and have been used in subsequent outbreaks [13, 14]. Likewise, the development and use of rapid and point-of-care tests were crucial for quickly diagnosing and isolating infected individuals, thereby containing the virus’s spread and facilitating timely treatment [15]. This experience has informed current pandemic preparedness by highlighting the necessity of global collaboration in research and development, swift regulatory processes and the establishment of robust clinical trials frameworks. Continued investment in research and development is crucial to stay ahead of emerging infectious diseases. Public-private partnerships play a significant role in ensuring that medical countermeasures are developed, tested, and made available to all quickly and in an equitable manner.

## Coordination

The Ebola crisis underscored the necessity of international collaboration and coordination. The global response involved multiple organizations, including the national Ministries of Health and governments of Liberia, Guinea and Sierra Leone, scientists, researchers, clinicians, public health officials and numerous government and non-governmental agencies. However, the response was initially fragmented. Streamlining coordination, sharing resources, and ensuring efficient logistics are imperative for a unified and effective response. The establishment of the WHO’s Health Emergencies Programme and the Coalition for Epidemic Preparedness Innovations (CEPI) in the aftermath of the outbreak were important steps towards better coordination of international health efforts for future emergency events.

The Ebola outbreak of 2014–2016 was a wake-up call for the global community, highlighting the vulnerabilities in our health systems and the necessity for comprehensive preparedness strategies. Ten years later, the lessons learned have led to significant advancements in collaborative surveillance, safe and scalable care, community protection, access to countermeasures and global coordination. The recent COVID-19 pandemic and global mpox outbreaks serve as stark reminders that preparedness is an ongoing process that requires sustained effort, investment, and global cooperation. As we move forward, building on the lessons from the 2014 West Africa Ebola outbreak and our more recent health emergencies will be essential in safeguarding against future health threats and ensuring a resilient global health system.
